# Comparative study of chemical treatments in combination with extrusion for the partial conversion of wheat and sorghum insoluble fiber into soluble

**DOI:** 10.1002/fsn3.1041

**Published:** 2019-04-30

**Authors:** Huma Bader Ul Ain, Farhan Saeed, Muhammad Asif Khan, Bushra Niaz, Samreen Gul Khan, Faqir Muhammad Anjum, Tabussam Tufail, Shahzad Hussain

**Affiliations:** ^1^ Institute of Home and Food Sciences Government College University Faisalabad Punjab Pakistan; ^2^ University of Agriculture Punjab Pakistan; ^3^ Department of Chemistry Government College University Faisalabad Punjab Pakistan; ^4^ The University of the Gambia Gambia; ^5^ College of Food and Agricultural Sciences King Saud University Riyadh Saudi Arabia

**Keywords:** chemical treatments, dietary fiber, extrusion, sorghum, wheat

## Abstract

Dietary fiber has gained greater attention owing to their positive and potential health perspectives. Cereals are the most important and enriched source of dietary fiber with more insoluble dietary fiber than soluble. For dietary fiber modification, chemical treatment with various techniques is considered as significant approach owing to its safety point of view and involves less damage to the molecular structure of the dietary fiber through chemical reagents and content of soluble dietary fiber is increased more efficiently. The current study was aimed to nutritionally characterize the cereal grains and to partially convert insoluble dietary fiber into soluble dietary fiber through chemical treatments in combination with extrusion. For the purpose, two varieties of each cereal were characterized for their chemical composition, mineral profile, and dietary fiber content according to the respective methods. Then, dietary fiber ratio in cereals was modified through chemical treatments, that is, acid, alkaline, and consecutive acid–alkaline followed by extrusion. Results regarding dietary fiber content of cereal grains exhibited that wheat (12.03–12.20 g/100 g) contained higher total dietary fiber followed by sorghum (6.70–6.90 g/100 g). Additionally, modification of SDF (1.97%) and IDF (11.48%) ratio in wheat and SDF (1.19%) and IDF (24.25%) ratio in sorghum through extrusion processing was nonsignificant while acid–alkaline treatment showed highly significant results, that is, 768.2% increase in SDF and 56.5% decrease in IDF in wheat and 952.38% increase in SDF and 71.17% decrease in IDF in sorghum. Among chemical treatments, higher result was given by acid–alkaline method and the lower outputs were observed in case of extrusion in both cereals. Conclusively, soluble dietary fiber was significantly increased through chemical treatments alone or in combination with twin‐screw extrusion.

## 1 INTRODUCTION

In recent era, the trend for dietary fiber consumption and utilization is increasing day by day owing to its functional properties (Lorencia & Alvarez, 2016; Singh, 2016). Dietary fiber is a nondigestible carbohydrate that is not absorbed in the largest part of alimentary canal (Park, Subar, Hollenbeck, & Schatzkin, 2011). Conventionally, based on solubility in water, dietary fiber is classified into two major classes including soluble dietary fiber (mainly pentosans, pectin, gums, and mucilage) and insoluble dietary fiber (cellulose, part of hemicellulose, and lignin) (Fuller, Beck, Salman, & Tapsell, 2016; Esposito et al., 2005; Borderias, Alonso, & Mateos, 2005). About contents of insoluble and soluble dietary fibers in foods, mainly cereals are 65%–80% and 20%–35%, respectively. Furthermore, recommended daily intake ratio for soluble and insoluble dietary fiber is 1:3, respectively (I & 3 quarters for soluble and insoluble dietary fiber). Among both classes of dietary fiber, soluble dietary fibers are more important with respect to functional and physiological perspectives than insoluble dietary fibers. The reason behind higher functionality of soluble dietary fiber is their rapid fermentation and breakdown in short‐chain fatty acids and higher consumption by the probiotics than insoluble fractions. Soluble dietary fiber has hypocholesterolemic effect due to the binding of soluble dietary fiber to the cholesterol and sugar, which lowers their absorption and transfer in the plasma (Abutair, Naser, & Hamed, 2018; McRae, 2018). Moreover, these fibers maintain the level of blood glucose and provide heart protection. These fibers have great role in weight management owing to their middle whittling perspective. Furthermore, soluble dietary fiber can pass easily through gastrointestinal tract through softness of stools, while insoluble dietary fibers do not solubilize in water and pass rapidly through gastrointestinal tract by providing bulk to the waste and preventing constipation and hemorrhoids.

Because of these positive and potential health perspectives, trend of fiber utilization in food products is increasing day by day (Brouns, Hemery, Price, & Anson, 2012). As the food applications of dietary fiber are concerned, it can be used in different nutritional products such as beverages, meat, drinks, and bakery products. The principal sources of dietary fibers are cereals, nuts, fruits, and vegetables. Among various sources of dietary fibers, the most important and enriched source of dietary fiber is cereals. Cereal is a prevalent harvest all over the mild and humid areas of the world from the family Gramineae. Major cereals are wheat, rice, rye, oat, sorghum, barley, and maize. These cereals provide 50% of the food energy worldwide (Awika, 2011). Wheat contains about 12% dietary fiber, whereas sorghum contains about 6% dietary fiber. For the partial conversion of insoluble dietary fiber into soluble dietary fiber, chemical treatment with various techniques is considered as significant approach. Among all conversion methods, chemical treatment is more safe owing to less damage to the molecular structure of the dietary fiber through chemical reagents and content of soluble dietary fiber is increased more efficiently. Chemical methods use acid and alkali to solubilize the dietary fiber of cereals. The main factors involved in this treatment are amount of acid and alkali, temperature and reaction time (Huang, He, Zou, & Liu, 2015).

Englyst and Cummings (1984) used sulfuric acid and trifluoroacetic acid to hydrolyze hemicellulose. This treatment hydrolyzes the long polysaccharide chain to smaller fractions to increase their solubility. Moreover, in a study, soluble dietary fiber content in black soybean hull was increased by using hydrogen peroxide (Feng et al., 2017). Furthermore, the dietary fiber ratio of whole grain barley was modified by carboxymethylation (Park, Lee, & Lee, 2013). Along with chemical treatments, another approach for dietary fiber modification is extrusion. Huang and Ma (2016) applied high temperature, pressure, and shear force to gasify and extend the moisture content present in the cereals. This mechanism depends upon the processing parameters such as temperature and pressure (Chen, Ye, Yin, & Zhang, 2014; Rashid, Rakha, Anjum, Ahmed, & Sohail, 2015). In general, a combined method may have greater effect on the modification of insoluble dietary fiber into soluble dietary fiber in cereal than the use of the single method (Ma & Mu, 2016; Tang et al., 2016).

Keeping in mind all the above‐mentioned views, there is a dire need to partially convert this insoluble dietary fiber into soluble dietary fiber and to develop the soluble dietary fiber‐enriched value‐added wheat and sorghum products. The objective of current study was to evaluate the effect of chemical treatment and extrusion on the modification of insoluble dietary fiber into soluble dietary fiber in two wheat and sorghum varieties.

## 2 MATERIALS AND METHODS

### 2.1 Procurement of raw material

Two varieties for each cereal, that is, wheat (Ujala‐16, FSD‐08) and sorghum (Sorghum‐11, JS‐02), were procured from Ayub Agriculture Research Institute (AARI), Faisalabad.

### 2.2 Chemical composition

The cereal grains were analyzed for moisture, crude fiber, crude protein, crude fat, ash content, and nitrogen‐free extract (NFE) (AOAC, 2005).

### 2.3 Mineral profile

The macrominerals (Ca, Mg, Na, K) and microminerals (Fe, Cu, Zn, Mn) of cereal grains were assessed according to the method of AOAC (1990).

### 2.4 Dietary fiber content

Total, soluble, and insoluble dietary fibers of cereal grains were analyzed by followed the principles of AACC (2000) method No. 32‐05.

### 2.5 Chemical treatments of cereal fiber

Dietary fiber from wheat and sorghum varieties was chemically modified and extruded for the partial conversion of insoluble dietary fiber into soluble dietary fiber according to the method of Ning, Villota, and Artz (1991). In brief, a mixture of wheat and sorghum fiber and water, at a ratio of 1:5, was used. Its pH values were adjusted acidic (pH 2.0–4.0) and alkaline (pH 9.0–11.0) by using 6.0N HCl (acid treatment) and 6.0N NaOH (alkaline treatment), respectively. After pH adjustment, the mixtures were then heated at 90°C for different periods of time ranging from 1 to 4 hr. At the end of each treatment, the supernatant was removed, neutralized, and then centrifuged at 700 *g* for 10 min. Pentose and hexose contents were measured for the acid treatment using high‐performance liquid chromatography, and total sugar content was determined for the alkaline treatment according to the procedure of Folkes and Taylor (1982) and Dubois et al. (1956), respectively. The precipitate was washed with water and dried using an air drier (Proctor Schwartz, Philadelphia, PA) at 75°C for 2 hr, followed by grinding and screening through a 2‐mm sieve. The acid–alkaline treatment involved consecutive acid and alkaline treatments, according to the aforementioned procedures. For the acidic treatment, the mixture was adjusted to pH 2.0 with 6.0N HCl, while for the alkaline treatment, the mixture was adjusted to pH 11.0 with 6.0N NaOH.

### 2.6 Extrusion of native and chemically modified cereal fiber

Native, and acid‐ and alkaline‐treated wheat and sorghum fiber were extruded using a ZSK‐30 twin‐screw extruder (Werner and Pfleiderer Crop., Ramsey, NJ) according to the Ning et al. (1991). Conditions of extrusion were selected according to preliminary work to ensure conditions suitable for bran modification. The barrel temperatures of the first two sections were maintained at 40 and 90°C, and the remaining three sections were held at 120°C. Extrusion was carried out at 50% moisture and a dry feed rate of 200 g/min. The screw speed used was 350 rpm. A dual‐orifice die (3 mm in diameter) was used. The extrudate was gently dried in an air dryer at room temperature for 24 hr, and then grounded and sieved.

### 2.7 Statistical analysis

The data obtained for each parameter were subjected for completely randomized design (CRD) and Latin square design (LSD) and later on ANOVA to determine the level of significance (Steel, Torrie, & Dickey, 1997).

## 3 RESULTS AND DISCUSSION

### 3.1 Chemical composition of wheat and sorghum

Table 1 exhibited mean values for moisture, ash, crude fat, crude fiber, crude protein, and nitrogen‐free extract in two different varieties. The results explicated that the higher moisture, crude fiber, and crude fat contents were shown by FSD‐08, whereas the higher ash, crude protein, and nitrogen‐free extract contents were exhibited by Ujala‐16. In Ujala‐16, moisture, ash, crude fat, crude protein, crude fiber, and nitrogen‐free extract were 9.7 ± 0.04, 1.7 ± 0.02, 1.9 ± 0.03, 10.6 ± 0.03, 1.9 ± 0.25, and 74.3 ± 0.32 g/100 g and in FSD‐08, 9.78 ± 0.03, 1.60 ± 0.03, 1.95 ± 0.04, 10.59 ± 0.05, 2.5 ± 0.4, 73.57 ± 0.36 g/100 g, respectively. These results are according to Lee, Nam, and Kong (2016) who observed the variability in chemical composition of wheat grain from different origins and found that moisture, crude protein, crude fat, crude fiber, and ash contents in wheat grain were 11.23%, 11.52%, 1.70%, 2.49%, and 1.54%, respectively.

**Table 1:** Mean values of chemical composition and mineral profile of wheat varieties

Wheat varieties	Chemical composition (%)						Macrominerals (g/kg)				Microminerals (g/kg)			
	Moisture	Ash	Crude fat	Crude protein	Crude fiber	NFE	K	Mg	Ca	P	Cu	Mn	Fe	Zn
Ujala‐16	9.7 ± 0.04^c^	1.7 ± 0.02^d^	1.9 ± 0.03^d^	10.6 ± 0.03^b^	1.9 ± 0.25^d^	74.3 ± 0.32^a^	322.12 ± 0.04^a^	141.17 ± 0.07^c^	35.44 ± 0.03^d^	291.00 ± 3.61^b^	0.16 ± 0.04^d^	2.09 ± 0.06^c^	4.07 ± 0.35^a^	3.07 ± 0.05^b^
FSD‐08	9.78 ± 0.03^c^	1.60 ± 0.03^e^	1.95 ± 0.04^e^	10.59 ± 0.05^b^	2.5 ± 0.4^d^	73.57 ± 0.36^a^	322.03 ± 0.02^b^	141.41 ± 0.05^c^	35.27 ± 0.04^d^	341.00 ± 6.56^a^	0.39 ± 0.04^a^	1.68 ± 0.04^d^	3.83 ± 0.40^b^	3 ± 0.85^b^
Mean	9.74	1.65	1.925	10.595	3.85	73.935	322.075	141.29	35.355	316	0.275	1.885	3.95	3.035

Note

Means carrying same letter are significantly identical.

Mean values revealed that Sorghum‐11 contained ash (1.6 ± 0.25%), crude protein (8.4 ± 0.31%), and nitrogen‐free extract (71.4 ± 1.12%) while JS‐02 comprised of ash (1.5 ± 0.40%), crude protein (7.5 ± 0.38%), and nitrogen‐free extract (70.6 ± 1.10%). Moreover, Sorghum‐11 contained lower amount of moisture (11.5 ± 0.35%), crude fat (3.6 ± 0.31%), and crude fiber (3.6 ± 0.30%) content while JS‐02 comprised of 12.2 ± 0.46% moisture, 4.0 ± 0.31% crude fat, and 4.1 ± 0.35% crude fiber contents (Table 2). The present investigation results are in line with the study of Palavecino, Penci, Dominguez, and Ribotta (2016) who worked on the proximate composition of sorghum flour and noticed that sorghum grain flour contained 12.21%, 3.67%, 3.90%, and 0.68% of crude protein, crude fat, crude fiber, and ash. In addition, Okoye and Obi (2017) evaluated that sorghum contained 9.68 ± 0.28% moisture, 7.84 ± 1.18% crude protein, 0.82 ± 0.04% fat, 0.64 ± 0.05% ash, and 1.66 ± 0.08% crude fiber.

**Table 2:** Mean values of chemical composition and mineral profile of sorghum varieties

Sorghum varieties	Chemical composition (%)						Macrominerals (g/kg)				Microminerals (g/kg)			
	Moisture	Ash	Crude fat	Crude protein	Crude fiber	NFE	K	Mg	Ca	P	Cu	Mn	Fe	Zn
Sorghum‐11	11.5 ± 0.35^b^	1.6 ± 0.25^e^	3.6 ± 0.31^d^	8.4 ± 0.31^c^	3.6 ± 0.30^d^	71.4 ± 1.12^a^	391.9 ± 0.45^a^	201.3 ± 0.16^c^	24.2 ± 0.31^d^	256.3 ± 0.14^b^	1.0 ± 0.04^d^	2.6 ± 0.06^c^	11.6 ± 0.31^a^	3.4 ± 0.56^a^
JS‐02	12.2 ± 0.46^b^	1.5 ± 0.40^e^	4.0 ± 0.31^d^	7.5 ± 0.38^c^	4.1 ± 0.35^d^	70.6 ± 1.10^a^	393.5 ± 0.40^a^	199.7 ± 0.32^c^	23.5 ± 0.31^d^	259.2 ± 0.26^b^	0.9 ± 0.03^c^	2.7 ± 0.05^b^	10.9 ± 0.40^b^	2.8 ± 0.31^b^
Mean	11.85	1.55	3.8	7.95	3.85	71	392.7	200.5	23.85	357.75	0.95	2.65	11.25	3.1

Note

Means carrying same letter are significantly identical.

### 3.2 Mineral profile of wheat and sorghum

Table 1 shows mean values for macromineral profile of two different wheat varieties, and these values revealed that highest potassium content (322.12 ± 0.04 g/kg) was observed in Ujala‐16 followed by FSD‐08 that was 322.03 ± 0.02 g/kg. Moreover, FSD‐08 revealed 141.41 ± 0.05 g/kg magnesium contents as compared to Ujala‐16 that was 141.17 ± 0.07 g/kg. Additionally, means for calcium contents revealed that Ujala‐16 has the highest content (35.44 ± 0.03 g/kg) followed by FSD‐08 (35.27 ± 0.04 g/kg). Furthermore, maximum phosphorus content was observed in FSD‐08 (341.00 ± 6.56 g/kg) as compared to Ujala‐16 (291.00 ± 3.61 g/kg). Mean values regarding microminerals have been depicted in Table 1 that revealed that Ujala‐16 was highest in manganese (2.09 ± 0.06 g/kg), iron (4.07 ± 0.35 g/kg), and zinc (3.07 ± 0.05 g/kg) contents, whereas FSD‐08 was on top position in copper (0.39 ± 0.04 g/kg) content. Moreover, manganese, iron, and zinc contents in FSD‐08 were 1.68 ± 0.04, 3.83 ± 0.40, and 3 ± 0.85 g/kg and copper content in Ujala‐16 was 0.16 ± 0.04 g/kg. The results are in line with the observation of Choi, Kang, Hyun, Lee, and Park (2013) who reported that wheat contained calcium, phosphorus, magnesium, potassium, copper, zinc, manganese, and iron in range of 39.76, 357.02, 126.88, 346.64, 0.41, 5.05, 2.43, and 4.48 mg/100 g, respectively.

Mean values regarding macromineral profile of two different sorghum varieties have been depicted in Table 2. According to these values, in Sorghum‐11, potassium, magnesium, calcium, and phosphorus were 391.9 ± 0.45, 201.3 ± 0.16, 24.2 ± 0.31, and 256.3 ± 0.14 g/kg, while 393.5 ± 0.40, 199.7 ± 0.32, 23.5 ± 0.31, and 259.2 ± 0.26 g/kg in JS‐02, respectively. Moreover, Table 2 exhibited microminerals for both sorghum varieties and results revealed that Sorghum‐11 was on highest position for copper (1.0 ± 0.04 g/kg), iron (11.6 ± 0.31 g/kg), and zinc (3.4 ± 0.56 g/kg) contents and at lower position for manganese (2.6 ± 0.06 g/kg), while JS‐02 was on lowest position for copper (0.9 ± 0.03 g/kg), iron (10.9 ± 0.40 g/kg), and zinc (2.8 ± 0.31 g/kg) contents and on top position for manganese (2.7 ± 0.05 g/kg) content. The present data regarding to macro‐ and microminerals of sorghum flour are corroborated with the study of Jimoh and Abdullahi (2017) who evaluated different varieties of sorghum flour for mineral profile and observed that sorghum flour is rich in magnesium (43.57 μg/g) followed by zinc (27.62 μg/g), calcium (17.58 μg/g), iron (13.03 μg/g), and copper (1.9 μg/g).

### 3.3 Dietary fiber of wheat and sorghum

Table 3 depicts means and standard deviations of soluble, insoluble, and total dietary fiber content of both wheat varieties. Results revealed that Ujala‐16 contained maximum soluble (3.05 ± 0.24%), insoluble (9.15 ± 0.17%), and total dietary fiber (12.20 ± 0.23%), whereas FSD‐08 comprised of lower soluble (3.02 ± 0.42%), insoluble (9.02 ± 0.30%), and total dietary fiber (12.03 ± 0.25%) contents, respectively. Wang, Klopfenstein, and Ponte (1993) probed the dietary fiber content of wheat grain and found that wheat grain contains about 1.25% soluble dietary fiber, 12.8% insoluble dietary fiber, and 14.0% total dietary fiber. Results are in accordance with the results of Ramulu and Rao (1997) who explicated that soluble, insoluble, and total dietary fiber in wheat flour were 2.47%–3.37%, 8.69%–10.63%, and 10.10%–12.00%.

**Table 3:** Mean values for dietary fiber content (g/100 g) of chemically treated wheat and sorghum varieties

Treatments	Wheat						Sorghum					
	Ujala‐16			FSD‐08			Sorghum−11			JS−02		
	SDF	IDF	TDF	SDF	IDF	TDF	SDF	IDF	TDF	SDF	IDF	TDF
Control	3.05	9.15	12.20	3.02	9.02	12.03	1.68	5.03	6.71	1.73	5.18	6.86
Extruded	3.11	8.10	11.21	3.14	8.05	11.19	1.70	6.25	7.95	1.80	6.27	8.07
Acid‐treated	4.25	7.58	11.83	4.45	7.56	12.00	2.76	3.62	6.38	3.10	3.78	6.88
Acid‐treated‐extruded	5.55	8.74	14.29	6.02	9.54	15.55	5.60	8.68	14.28	6.09	9.59	15.58
Alkaline‐treated	9.88	8.48	18.36	9.93	8.44	18.37	9.88	8.41	18.29	11.27	8.35	19.62
Alkaline‐treated‐extruded	5.61	8.70	14.31	6.12	9.44	15.56	5.69	8.70	14.3	6.15	9.48	15.63
Alkaline–acid‐treated	8.12	8.25	16.37	8.90	9.74	18.64	8.12	8.25	16.31	8.99	9.83	18.82
Acid–Alkaline‐treated	26.48	3.98	30.46	24.28	4.08	28.36	39.62	2.50	42.12	49.96	2.64	49.60
Means	8.26^a^	7.87^a^	16.13^a^	8.23^a^	8.12^a^	16.35^a^	5.94^a^	4.25>^b^	10.19^a^	5.98^a^	4.44^a^	10.42^a^

Note

Means carrying same letters are significantly identical.

Abbreviation(s): IDF, insoluble dietary fiber; SDF, soluble dietary fiber; TDF, total dietary fiber.

Mean values for both sorghum varieties related to dietary fiber are shown in Table 3. Results explicated that higher soluble (1.68 ± 0.01%), insoluble (5.025 ± 0.01%), and total dietary fiber (6.70 ± 0.17%) contents were shown by JS‐02 and lower soluble (1.725 ± 0.01%), insoluble (5.175 ± 0.01%), and total dietary fiber (6.90 ± 0.45%) contents were shown by Sorghum‐11. Sorghum contained 26.34 ± 0.13 g/100 g total dietary fiber, 25.37 ± 0.27 g/100 g insoluble dietary fiber, and 0.97 ± 0.14 g/100 g soluble dietary fiber (Moraes et al., 2015).

### 3.4 Partial conversion of IDF into SDF in wheat through chemical treatments

Table 3 shows the mean values of dietary fiber content in chemically modified varieties of wheat. It was revealed from results of extrusion that the ratio of soluble and insoluble dietary fibers of both varieties modified slightly, that is, soluble dietary fiber was slightly increased and insoluble dietary fiber was slightly decreased in both barley varieties. In Ujala‐16, soluble dietary fiber was increased from 3.05 to 3.11 g/100 g and insoluble dietary fiber decreased from 9.15 to 8.10 g/100 g, whereas in FSD‐08, increase in soluble dietary fiber was from 3.02 to 3.14 g/100 g and decrease in insoluble dietary fiber was from 9.02 to 8.05 g/100 g. These results showed that modification of soluble (1.97%) and insoluble fiber (11.48%) ratio was more in FSD‐08 than in soluble (3.97%) and insoluble fiber (10.75%) ratio of Ujala‐16. However, the overall modification was not significant (*p* > 0.05).

**Figure 1:**
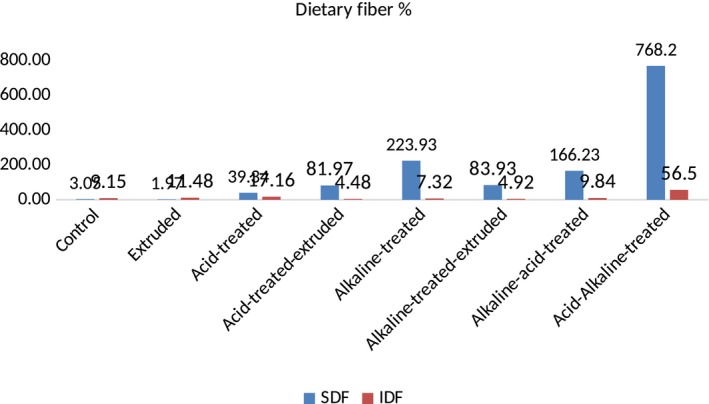
DF content of chemically modified Ujala‐16

**Figure 2:**
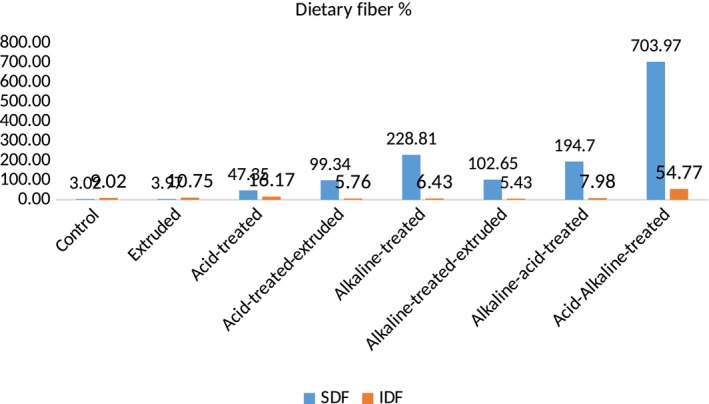
DF content of chemically modified FSD‐08

Moreover, when the dietary fiber was treated by simply by acid, it significantly modified the soluble (4.25 g/100 g) and insoluble (7.58 g/100 g) dietary fiber ratio, that is, 39.34% increase in soluble and 17.16% decrease in insoluble dietary fiber in Ujala‐18, while in FSD‐08, 47.35% increase in soluble and 16.17% decrease in insoluble dietary fiber, respectively. After treatment with acid, dietary fiber of both wheat varieties was then extruded (simultaneous treatments) and resulted modification of dietary fiber was much better, that is, in Ujala‐16, soluble dietary fiber was increased from 3.05 to 5.55 g/100 g and insoluble dietary fiber was decreased from 9.15 to 7.58 g/100 g, whereas in FSD‐08, increase in soluble dietary fiber was from 3.02 to 6.02 g/100 g and decrease in insoluble dietary fiber was from 9.02 to 8.54 g/100 g. Moreover, graphical representation regarding acid‐treated and extruded wheat dietary fiber showed 81.97% increase in soluble and 4.48% decrease in insoluble dietary fiber in Ujala‐16, whereas 99.34% increase in soluble and 5.76% decrease in insoluble dietary fiber in FSD‐08 as shown in Figures 1, 2.

Furthermore, after acid and acid–extrusion treatment, dietary fibers from both wheat varieties were treated with alkali (NaOH). This treatment significantly modified the soluble and insoluble ratio. In Ujala‐16, soluble dietary fiber was significantly increased from 3.05 to 9.88 g/100 g and insoluble dietary fiber was significantly decreased from 9.15 to 8.48 g/100 g, while in FSD‐08, soluble dietary fiber was significantly increased from 3.02 to 9.93 g/100 g and insoluble dietary fiber was significantly decreased from 9.02 to 8.44 g/100 g. Then, this alkali‐treated dietary fiber was extruded, and the results were significant (*p* < 0.05). Soluble dietary fiber was increased from 3.05 to 5.61 and from 3.02 to 6.12 g/100 g, while insoluble dietary fiber was decreased from 9.15 to 8.70 and 9.02 to 9.44 g/100 g in Ujala‐16 and FSD‐08, respectively.

After that, alkaline treatment and acid treatment were applied simultaneously in two sequences (i.e., alkaline–acid treatment and acid–alkaline treatment). The results of acid and alkaline treatments were significant (*p* < 0.05). Alkaline–acid treatment increased soluble dietary fiber from 3.05 to 8.12 g/100 g and from 3.02 to 8.90 g/100 g and decreased insoluble dietary fiber from 9.15 to 8.25 g/100 g and from 9.02 to 9.74 in Ujala‐16 and FSD‐08, respectively. Whereas, acid–alkaline treatment increased the soluble dietary fiber from 3.05 to 26.48 g/100 g (768.2%) whereas decrease in insoluble dietary fiber was from 9.15 to 3.98 g/100 g (56.5%) in Ujala‐16 while, in FSD‐08, soluble dietary fiber was increased from 3.02 to 24.28 g/100 g (703.97%) and insoluble dietary fiber was decreased from 9.02 to 4.08 g/100 g (54.77%).

Keeping in view all these results, acid–alkaline treatment showed highest results in terms of modification of dietary fiber than all other treatments. In all techniques, breakdown of large polysaccharides into smaller oligosaccharide fractions occurred which was resulted in increase in solubility of these smaller fractions. In case of chemical treatments, this modification was occurred owing to the different chemicals such as alkali and acid, while in case of extrusion, high temperature, pressure, and shear force were applied to the polysaccharides and insoluble fractions of dietary fiber. These conditions gasified and extended the moisture content present in the both varieties of wheat. Moreover, mode of action of consecutive acid–alkaline treatments was that acid treatment can open the structure and increase the surface porosity of the fiber particle, making it easier for the hydroxyl groups to penetrate inside and perform hydrolysis during the subsequent alkaline treatment.

### 3.5 Partial conversion of IDF into SDF in sorghum through chemical treatments

Table 3 explicates the mean values of dietary fiber content in chemically modified varieties of sorghum. Among all treatments, highest results were shown by acid–alkaline treatment and lowest results were shown by extrusion. Acid–alkaline treatment highly modified the ratio of soluble and insoluble dietary fibers of both sorghum varieties, that is, soluble dietary fiber was highly increased and insoluble dietary fiber was highly decreased in both sorghum varieties. In Sorghum‐11, soluble dietary fiber was increased from 1.68 to 39.62 g/100 g and insoluble dietary fiber decreased from 5.03 to 2.50 g/100 g, whereas in JS‐02, increase in soluble dietary fiber was from 1.73 to 49.96 g/100 g and decrease in insoluble dietary fiber was from 5.18 to 2.64 g/100 g. These results showed that modification of soluble (952.38%) and insoluble fiber (71.17%) ratio was more in Sorghum‐11 than in soluble (786.71%) and insoluble fiber (62.55%) ratio of JS‐02.

**Figure 3:**
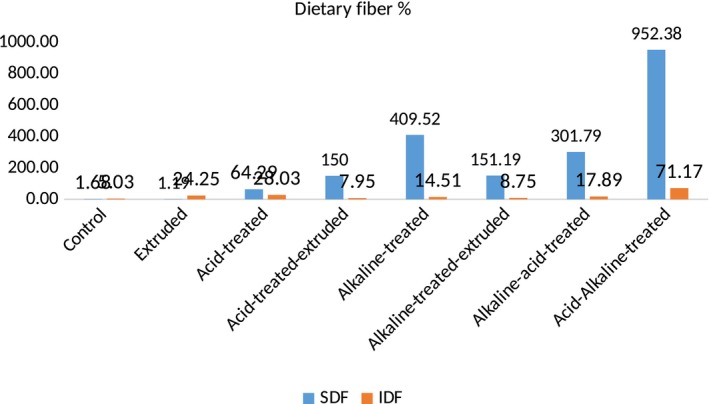
DF content of chemically modified Sorghum‐11

**Figure 4:**
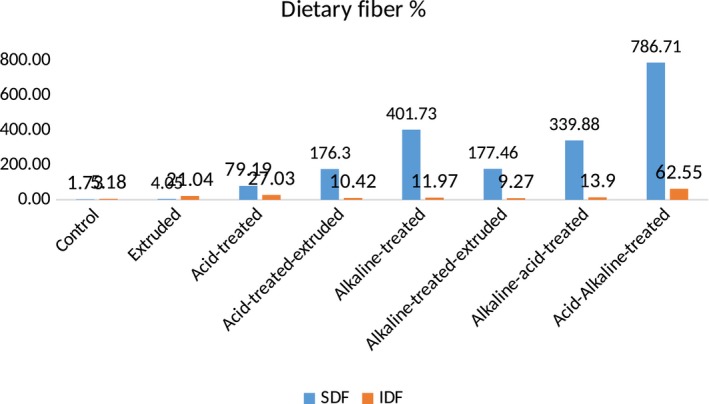
DF content of chemically modified JS‐02

However, extrusion cooking slightly modified the dietary fiber ratio of both sorghum varieties. It increased the soluble dietary fiber from 1.68 to 1.70 g/100 g (1.19%), whereas change in insoluble dietary fiber was from 5.03 to 6.25 g/100 g (24.25%) in Sorghum‐11, while in JS‐02, soluble dietary fiber was increased from 1.73 to 1.80 g/100 g (4.05%) and insoluble dietary fiber was decreased from 5.18 to 2.64 g/100 g (21.04%) (Figures 3, 4). Moreover, other techniques showed higher results than extrusion but lower than acid–alkaline method. When the sorghum dietary fiber was treated by simply by acid, it significantly modified the soluble (3.10 g/100 g) and insoluble (3.78 g/100 g) dietary fiber ratio, that is, 79.19% increase in soluble and 27.03% decrease in insoluble dietary fiber in JS‐02, while in Sorghum‐11, 64.29% increase in soluble (2.76 g/100 g) and 28.03% decrease in insoluble (3.62 g/100 g) dietary fiber, respectively.

After treatment with acid, dietary fiber of both sorghum varieties was then extruded (simultaneous treatments) and resulted modification of dietary fiber was much better, that is, in Sorghum‐11, soluble dietary fiber was increased from 1.68 to 5.60 g/100 g and insoluble dietary fiber was decreased from 5.03 to 8.68 g/100 g, whereas in JS‐02, increase in soluble dietary fiber was from 1.73 to 6.09 g/100 g and decrease in insoluble dietary fiber was from 5.18 to 9.59 g/100 g. Furthermore, after acid and acid–extrusion treatment, dietary fiber from both sorghum varieties was then treated with alkali (NaOH). This treatment significantly modified the soluble and insoluble ratio. In Sorghum‐11, soluble dietary fiber was significantly increased from 1.68 to 9.88 g/100 g and insoluble dietary fiber was significantly decreased from 5.03 to 8.41 g/100 g, while in JS‐02, soluble dietary fiber was significantly increased from 1.73 to 11.27 g/100 g and insoluble dietary fiber was significantly decreased from 5.18 to 8.35 g/100 g. Then, this alkali‐treated dietary fiber was extruded, and the results were significant (*p* < 0.05). Soluble dietary fiber was increased from 1.68 to 5.69 and from 1.73 to 6.15 g/100 g, while insoluble dietary fiber was decreased from 5.03 to 8.70 and 5.18 to 9.48 g/100 g in Sorghum‐11 and FSD‐08, respectively.

After that, alkaline–acid treatment was applied and showed significant results. This treatment increased soluble dietary fiber from 1.68 to 8.12 g/100 g and from 1.73 to 8.99 g/100 g and decreased insoluble dietary fiber from 5.03 to 8.25 g/100 g and from 5.18 to 9.83 in Sorghum‐11 and JS‐02, respectively. The mechanism behind this modification in dietary fiber through different chemical techniques and extrusion was much similar. All treatments modified the dietary fiber ratio of both varieties of sorghum significantly, highly significantly, or insignificantly. The mode of action of all treatments was almost same. All treatments break down insoluble larger fractions into soluble smaller fractions and solubilized them either through acid and alkali or through high temperature, pressure and shear force.

## 4 CONCLUSION

Both wheat and sorghum varieties were found to be rich source of protein and potassium. The ratio of dietary fiber in wheat and sorghum was modified through chemical treatments along with extrusion. All treatments significantly increased soluble dietary fiber and decreased insoluble dietary fiber but acid–alkaline treatment was highly significant. This modification opens the door for the betterment of physiochemical, physiological, and functional properties of dietary fiber by increasing the soluble dietary fiber. The trial should be conducted at industrial premises for partial conversion of insoluble dietary fiber into soluble dietary fiber to improve functional aspects of cereals. Novel products with improved soluble dietary fiber should be introduced in market. As good source of soluble dietary fiber, treated cereals can be incorporated in baking products to make functional various diseases because consumers are more conscious toward their diet and wish for natural remedies. Efficacy investigations should be conducted to verify the improvement in functional aspects of cereal dietary fiber against various maladies.

## CONFLICT OF INTEREST

Authors declare that they have no conflict of interest.

## ETHICAL APPROVAL

This article does not contain any studies with human participants or animals performed by any of the authors. It is further certified that human and animal testing is unnecessary in this study.

## INFORMED CONSENT

For this type of study, formal consent is not required.
